# Identification and genomic comparison of temperate bacteriophages derived from emetic *Bacillus cereus*

**DOI:** 10.1371/journal.pone.0184572

**Published:** 2017-09-08

**Authors:** Peiling Geng, Shen Tian, Zhiming Yuan, Xiaomin Hu

**Affiliations:** 1 Key Laboratory of Special Pathogens and Biosafety, Center for Emerging Infectious Diseases, Wuhan Institute of Virology, Chinese Academy of Sciences, Wuhan, China; 2 University of the Chinese Academy of Sciences, Beijing, China; Centro Nacional de Biotecnologia, SPAIN

## Abstract

Cereulide-producing *Bacillus cereus* isolates can cause serious emetic (vomiting) syndrome and even acute lethality. As mobile genetic elements, the exploration of prophages derived from emetic *B*. *cereus* isolates will help in our understanding of the genetic diversity and evolution of these pathogens. In this study, five temperate phages derived from cereulide-producing *B*. *cereus* strains were induced, with four of them undergoing genomic sequencing. Sequencing revealed that they all belong to the *Siphoviridae* family, but presented in different forms in their hosts. PfNC7401 and PfIS075 have typical icosahedral heads, probably existing alone as phagemids in the host with self-replicating capability in the lysogenic state. PfEFR-4, PfEFR-5, and PfATCC7953 have elongated heads, with the genomes of the former two identified as linear dsDNA, which could be integrated into the host genome during the lysogenic state. Genomic comparison of the four phages with others also derived from emetic *B*. *cereus* isolates showed similar genome structures and core genes, thus displaying host spectrum specificity. In addition, phylogenic analysis based on the complete genome and conserved tail fiber proteins of 36 *Bacillus* species-derived phages confirmed that the phages derived from emetic *B*. *cereus* strains were highly similar. Furthermore, one endolysin LysPfEFR-4 was cloned and showed lytic activity against all tested emetic *B*. *cereus* strains and cross-lytic activity against some other pathogenic bacteria, implying a potential to control bacterial contamination in the food supply.

## Introduction

*Bacillus cereus* is a common opportunistic pathogen causing clinical cases of food poisoning, i.e., diarrhea and emesis. The latter is associated to cereulide, a single heat-stable cyclic dodecadepsipeptide toxin [1]. As cereulide is toxic to mitochondria due to its action as a potassium ionophore and killer of human natural killer cells, it represents one of the most serious food safety risks of *B*. *cereus* [2]. The ecology and evolution of emetic *B*. *cereus* has, therefore, raised many concerns. Most cereulide-producing *B*. *cereus* isolates, even from different ecological niches, are relatively conserved at the chromosomal level, with previous study confining them to a single and homologous evolutionary lineage [3]. In addition, although rare, cereulide-producing *Bacillus weihenstephanensis* isolates with psychrotolerant features, which similarly belong to the *B*. *cereus* group, have also been identified [4]. The genomic variation at the chromosomal level between emetic *B*. *weihenstephanensis* and *B*. *cereus* is greater than that within the emetic *B*. *cereus* lineage. Furthermore, they both show flexible plasmid content [5], as well as different genomic locations (on chromosomes or plasmids) of cereulide biosynthesis gene clusters and their relatedness to mobile genetic elements (MGEs) [6]. In addition to plasmids, prophages represent another extrachromosomal material bearing adaptive genetic determinants and horizontal genetic transfer potential. The exploration of the prophages in emetic *B*. *cereus* strains will be helpful for better understanding the evolution and expansion of their ecological niches.

Prophages are ubiquitous in many kinds of bacteria, and result from the lysogenic integration of the genomes of temperate phages into their host bacterium’s chromosomes or plasmids. They can replicate themselves along with bacterial genomes or exist as an extrachromosomal plasmid. If harboring genes enough to produce a mature virion, the prophage can be excised from the bacterial genome and access the lytic cycle when exposed to UV light or certain chemical agents [7–9]. As a type of MGE, prophages significantly contribute to bacterial genetic diversity and endow the host with special characteristics, such as pathogenicity and distinct phenotypic traits through transduction, lysogenic conversion, or active lysogeny driving host evolution [10–13]. For instance, prophage ɸ11 can lead *Staphylococcus aureus* to acquire antibiotic resistance through autotransduction [14]. In addition, prophages Gifsy-1 and Gifsy-2 play important roles in the infection ability of *Salmonella enterica serovar* Typhimurium in mice by carrying virulence or immune-related genes that help their bacterial hosts defend against macrophages [15]. The A118-like prophage regulates *Listeria monocytogenes* to escape from mammalian cells phagosomes by reversible active lysogeny via insertion into or excision from the competence system master regulator *comK* gene [12]. Cyanobacterial cells differentiate into nitrogen-fixing cells along with the non-reversible excision of prophage elements from three nitrogen fixation process-related genes under nitrogen-starving conditions [12]. Based on their ubiquitous and unique features, prophages have been used for bacterial typing in *Escherichia coli*, *Streptococcus pneumonia*, and *Bacillus anthracis* [16–18]. However, despite a number of *B*. *cereus* group phages being isolated and sequenced, only limited information on emetic *B*. *cereus* phages and genomic data have been reported (e.g., Tp250, vB_BceS-IEBH, and 11143) [8, 19, 20], hindering further research on the coevolution and interaction of cereulide-producing *B*. *cereus* and its related phages.

In this study, we describe the general features of temperate bacteriophages originating from emetic *B*. *cereus* isolates and genomic insights resulting from comparative and functional gene analysis. Five bacteriophages were isolated and characterized. The whole genomes of four bacteriophages were sequenced and compared with those derived from *Bacillus* species isolates. One endolysin was cloned and characterized. This study will help in furthering our understanding of the temperate phages from emetic *B*. *cereus* isolates as well as provide a possible strategy to control this pathogen.

## Results

### Bacteriophage induction and morphological observation

**Fig 1:**
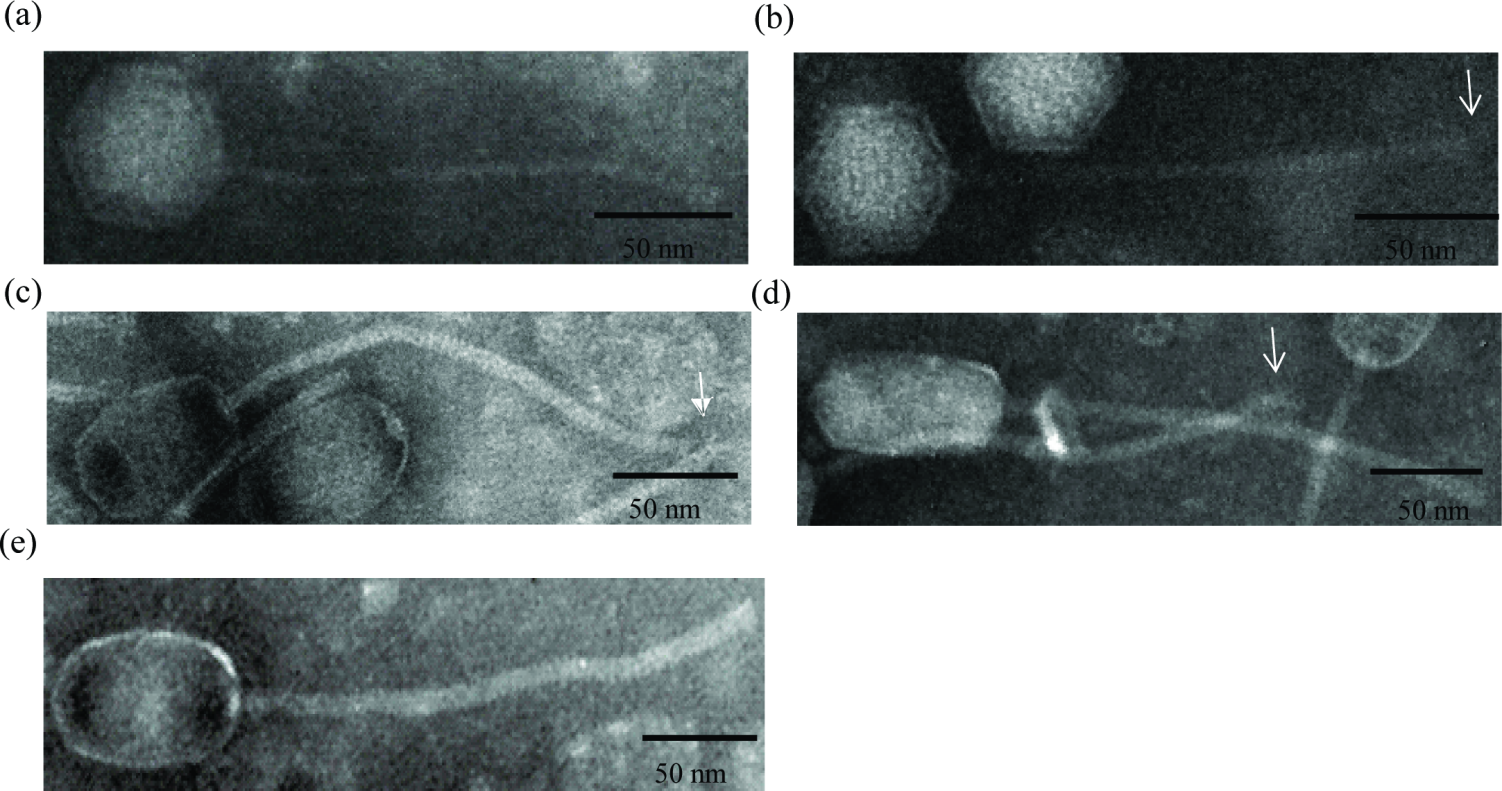
Transmission electron micrographs of phages isolated in this study. PfNC7401 (a), PfIS075 (b), PfEFR-4 (c), PfEFR-5 (d), and PfATCC7953 (e) were stained with 2% phosphotungstic acid and visualized. White arrow indicates the putative tail fiber. Scale bars are 50 nm.

**Fig 2:**
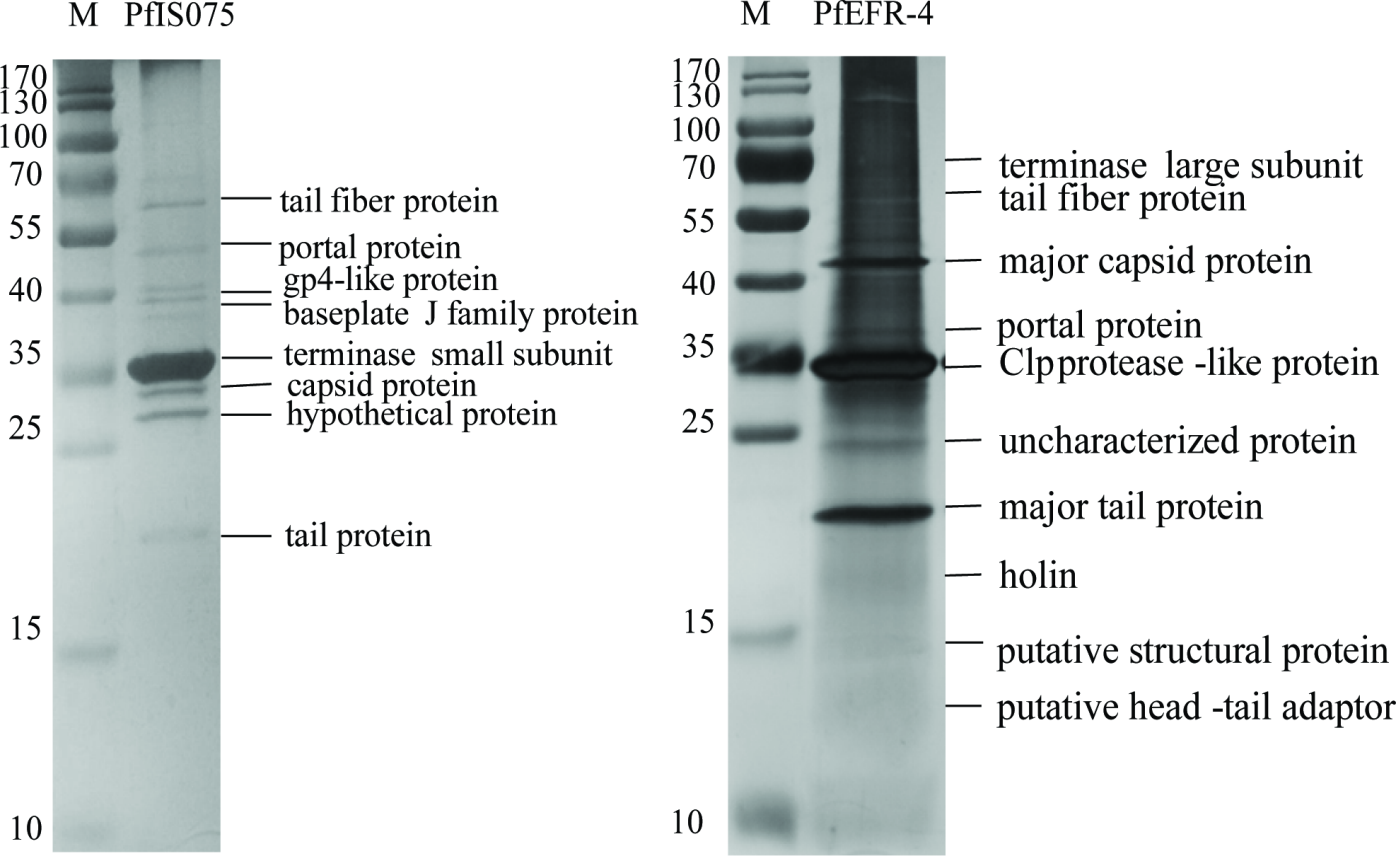
Proteomics analysis of the purified phages. Structural protein patterns of phages by 15% SDS-PAGE. Lane M: SM0671 protein marker. The PfIS075 and PfEFR-4 proteins identified by LC-MS/MS are shown on the right of the SDS-PAGE gels.

With the application of mitomycin C, bacterial lysis was observed during the growth of emetic *B*. *cereus* strains NC7401, IS075, EFR-4, EFR-5, and ATCC7953, indicating the induction of their prophages (S1 Fig). Furthermore, five phages, named PfNC7401, PfIS075, PfEFR-4, PfEFR-5, and PfATCC7953, were observed by transmission electron microscopy (TEM) from their concentrated lysates (Fig 1). All belonged to the *Siphoviridae* family in *Caudovirales*. Both PfNC7401 and PfIS075 had typical icosahedral heads (55.11−57.90 nm) and long non-contractile tails (2.9−3.5 nm × 170.83−70.95 nm), whereas PfEFR-4, PfEFR-5, and PfATCC7953 had elongated heads (44.32−57.66 nm × 88.61−100.23 nm) and long non-contractile tails (5.88−8.82 nm × 241.43−269.11 nm). The major structural protein profiles of PfNC7401 and PfIS075 were identical, as were those of PfEFR-4, PfEFR-5, and PfATCC7953 (data not shown). Therefore, the major structural proteins of PfIS075 and PfEFR-4 were identified through LC-MS/MS, and were found to contain a capsid protein, tail protein, tail fiber protein, DNA packaging protein such as portal protein, and some uncharacterized proteins, as listed in Fig 2.

### Host range and plaque formation

**Fig 3:**
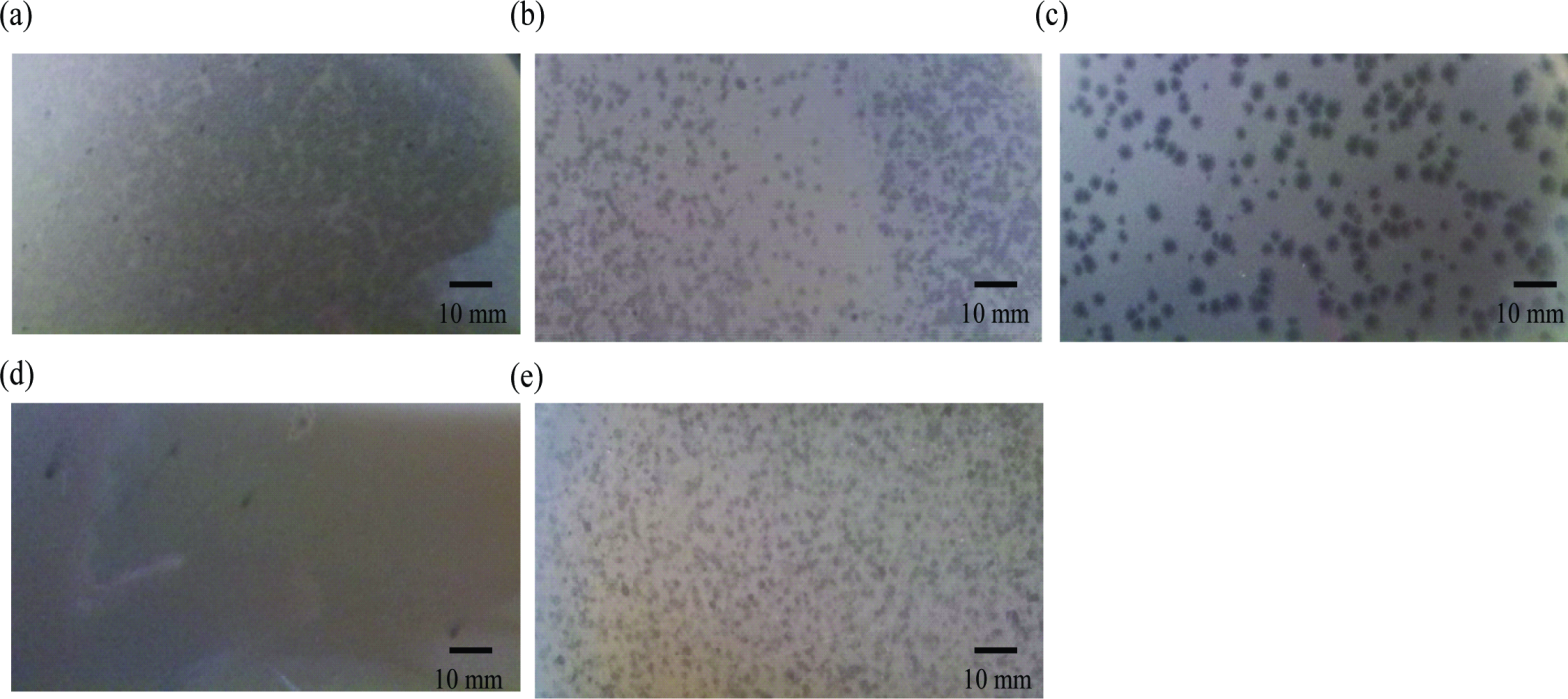
Plaques of temperate bacteriophages. Various plaques were formed on soft agar (0.5%) by PfNC7401 (a), PfIS075 (b), PfEFR-4 (c), PfEFR-5 (d), and PfATCC7953 (e) with their propagation strains. Scale bars are 10 mm.

The host range was evaluated by the lysis zone formed from a droplet of the purified phages (titer > 10^5^ PFU/mL) onto the bacterial lawns. The tested strains sensitive to PfNC7401, PfIS075, PfEFR-4, PfEFR-5, and PfATCC7953 all belonged to emetic *B*. *cereus*, except ATCC10987 and Schrouff (Table 1), indicating both species and strain specificity. The strain displaying the strongest sensitivity was used as the propagation host for the corresponding phage. PfNC7401, PfIS075, and PfATCC7953 formed small and clear plaques, whereas PfEFR-4 and PfEFR-5 formed large and clear plaques on their propagation host strains, respectively (Fig 3).

**Table 1:** Host range patterns of isolated bacteriophages.

Strains	Characteristics [21]	Origin	Accession number	Plaque forming ability ^a^				
				PfNC7401 (No. KX227758)	PfIS075 (No. KX227759)	PfEFR-4 (No. KX227757)	PfEFR-5 (No. KX227760)	PfATCC7953 (No. ND)
F4810/72	*B*. *cereus*, emetic	Vomit of a patient, UK	CP001177	++	-	-	-	-
NC7401	*B*. *cereus*, emetic	Emetic food poisoning, Japan	NC_016771	++	-	++	++	++
5975C	*B*. *cereus*, emetic	Food—pasta, Belgium	AHEL01000000	++	-	-	-	+
IS075	*B*. *cereus*, emetic	Animal—vole, Poland	AHCH00000000.2	-	+	-	-	-
LH001	*B*. *cereus*, emetic	Food, Belgium	ND	+++^b^	-	+	+	-
EFR-1	*B*. *cereus*, emetic	Food, China	ND	++	++	++	++	++
EFR-2	*B*. *cereus*, emetic	Food, China	ND	+	-	-	-	-
EFR-4	*B*. *cereus*, emetic	Food, China	ND	++	+	+++ ^b^	+++ ^b^	+++ ^b^
EFR-5	*B*. *cereus*, emetic	Food, China	ND	++	+++ ^b^	++	++	++
ATCC7953	*B*. *cereus*, emetic	Food, China	ND	-	-	-	-	-
AND1407	*B*. *cereus*, emetic	Food—blackcurrant, Denmark	AHCM01000000	-	-	-	-	-
CER057	*B*. *weihenstephanensis*, emetic	Food—parsley, Belgium	AHDS01000000	-	-	-	-	-
MC67	*B*. *weihenstephanensis*, emetic	Soil, Sweden	AHEN01000000	-	-	-	-	-
ISP2954	*B*. *cereus*	Food, Belgium	AHEJ01000000	-	-	-	-	-
ATCC10987	*B*. *cereus*	Cheese spoilage, Canada	AE017194	++	+	-	-	-
ATCC14579	*B*. *cereus*	Clinical isolate?	AE016877.1	-	-	-	-	-
Schrouff	*B*. *cereus*	Food, Belgium	AHCI00000000.1	-	+	-	-	-
ISP3191	*B*. *cereus*	Food—spice, Belgium	AHEK01000000	-	-	-	-	-
A16R	*B*. *anthracis*, vaccine strain	Vaccine strain, China	CP001974.2	-	-	-	-	-
HD-73	*B*. *thuringiensis* var *kurstaki*	ND	NC_020238.1	-	-	-	-	-
BMB171	*B*. *thuringiensis* cured of plasmids	Acrystalliferous mutant strain, China	NC_014171.1	-	-	-	-	-
ATCC35646	*B*. *thuringiensis* var *israelensis*	ND	ND	-	-	-	-	-
KBAB4	*B*. *weihenstephanensis*	Soil	NC_010184.1	-	-	-	-	-
DSMZ2048R	*B*. *mycoides*	ND	ND	-	-	-	-	-
168	*B*. *subtilis*	ND	NC_000964.3	-	-	-	-	-
C3-41	*L*. *sphaericus*	Mosquito breeding site, China	CP000817.1	-	-	-	-	-
RN4220	*S*. *aureus*	Mutant lab strain	NZ_AFGU00000000.1	-	-	-	-	-
O901	*S*. Typhimurium	Clinical diagnosis, China	ND	-	-	-	-	-

^a^Host range pattern of the phages was measured qualitatively and quantitatively by plaque forming ability, with (+++) for strongest lysis, (++) for moderate lysis, (+) for slight lysis, and (-) for no lysis.

^b^Strains were used as the propagation host for the corresponding phage.

No. indicates GenBank accession number of phages. ND means that the information was not determined.

### General features of the genomes of the four phages

The genomes of PfNC7401, PfIS075, PfEFR-4, and PfEFR-5 were sequenced. PfIS075 and PfNC7401 had a genome of 48,626 and 47,972 bp, both with ca. 36.5% GC content, encoding 81 and 79 putative open reading frames (ORFs), respectively. Alignments of the two phages and their host genomes, which were sequenced previously, were performed. The genome of PfNC7401 demonstrated a complete match to the sequence of pNC1 (Accession No. NC_016772.1) of *B*. *cereus* NC7401, which was originally annotated as a plasmid, and PfIS075 also matched accurately to one scaffold (Accession No. AHCH02000044.1) of the gapped genome of IS075 (data not shown). PfEFR-4 and PfEFR-5 had a genome of 43,223 and 43,773 bp, both with ca. 35.4% GC content, encoding 67 and 69 putative ORFs, respectively (S1 Table).

Restriction profiles (S2 Fig) and PCR results using the primers listed in S2 Table revealed that the genomic DNA of PfIS075 and PfNC7401 were circular, whereas PfEFR-4 and PfEFR-5 exhibited linear dsDNA (S3 Fig). All four phages had mini-satellite DNA, mainly related to DNA replication proteins (data not shown). No tRNA, rRNA, or sRNA were identified in the genomes. The genomes of PfEFR-4 and PfEFR-5 were almost identical, except that PfEFR-4 had only one integrase, whereas PfEFR-5 had one more (ORF31) upstream of the lysogeny control module. PfIS075 and PfNC7401 also shared great similarity, though differences were observed within two hypothetical proteins, of which PfIS075-ORF33 and PfNC7401-ORF44 had similar N-terminal sequences belonging to the DUF2479 superfamily encoding a putative long tail fiber, and PfIS075-ORF34 had no match in PfNC7401.

**Fig 4:**
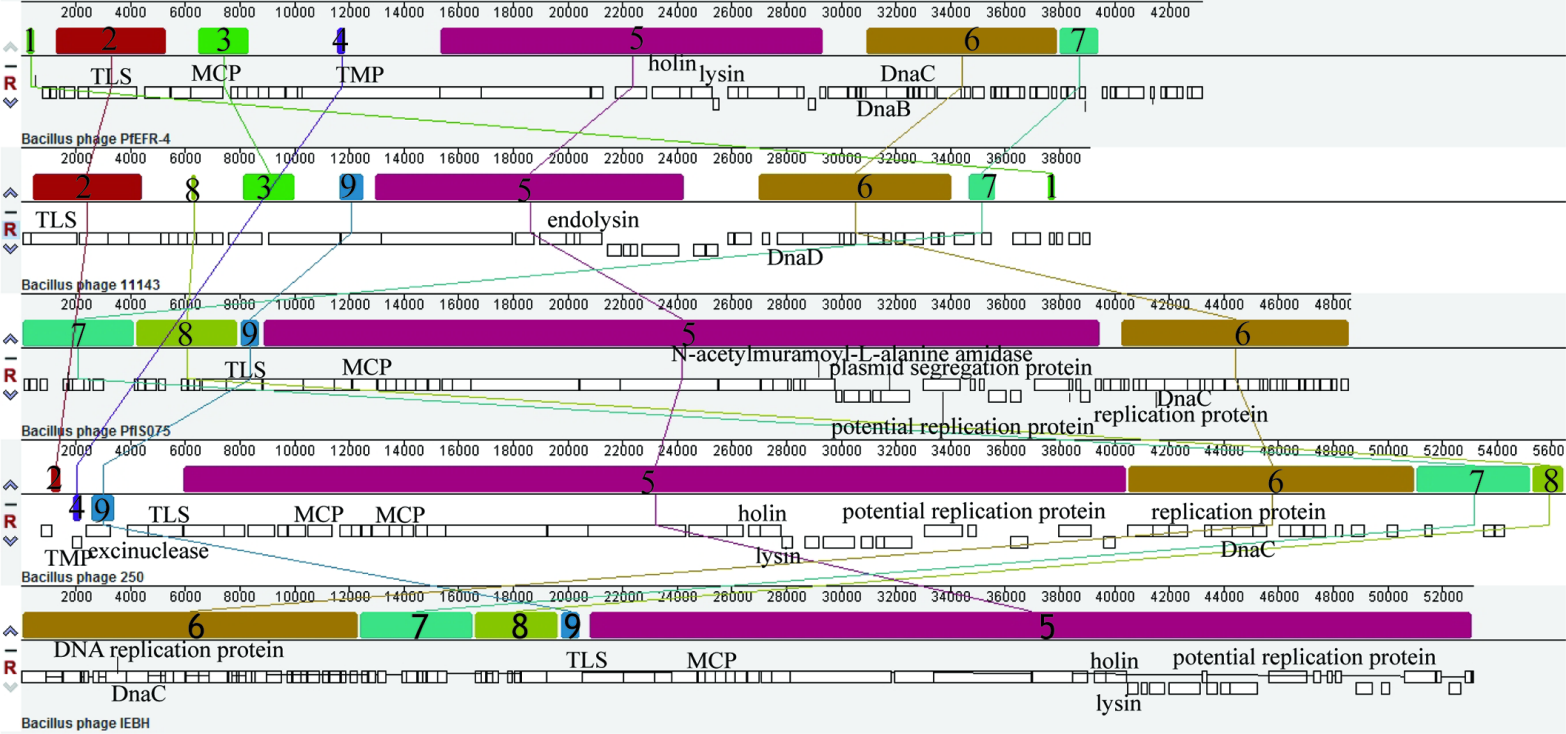
Comparative genome analysis of five bacteriophages derived from emetic *B*. *cereus* using the Mauve program. Genomes used for alignment were from phages PfEFR-4, 11143, PfIS075, Tp250, and vB_Bces-IEBH. The putative ORFs are presented as white rectangles and the nine LCBs are indicated as various contiguously colored regions. The genome of PfEFR-4 was used as a reference sequence.

The genomes of PfEFR-4 and PfIS075 were compared with three other emetic *B*. *cereus*-derived phages (vB_Bces-IEBH, Tp250, and 11143) and visualized using Mauve [22] (Fig 4). This revealed mosaic genome structures, exhibiting nine locally collinear blocks (LCBs). The LCBs 5−7 involved in structural proteins, host lysis, DNA replication, and transcriptional regulator genes showed good collinearity within the five phages, whereas the LCBs 8−9 involved in DNA packaging and LCBs 4 and 9 encoding tape measure protein (TMP) and exonuclease, respectively, displayed rearrangement and inversion. Furthermore, core gene analysis showed that virion morphogenesis-related genes, DNA replication and regulation genes, and lysis-related genes were similar among the five temperate phages derived from emetic *B*. *cereus* (data not shown).

Through analysis of the DNA packing modules, e.g., terminase large subunits (TLSs), like vB_BceS-IEBH, both PfIS075 and Tp250 showed putative headful packaging mechanisms, whereas PfEFR-4 and 11143 exhibited putative site-specific packaging mechanisms. The TLS of PfIS075 showed great similarity with that of the two headful packaging phages, ca. 92% similarity with the TLS of vB_BceS-IEBH (YP_002154374.1) and 80% with the TLS of A118 (NP_463463.1) [20, 23]. The TLS of PfEFR-4 and 11143 revealed 77% and 46% similarity, respectively, with that of *Paenibacillus* phage Harrison (YP_009193815.1), which has site-specific packaging with “cohesive ends with 3’ overhangs” [24]. The TLS of Tp250 shared 85% similarity with that of PBC1 (YP_006383455.1), which carries terminally redundant and partially permuted genomes [25], thus Tp250 might also have a headful packaging mechanism.

The major capsid protein (MCP) of PfIS075 (ORF21) revealed ca. 82% similarity with that of vB_BceS-IEBH (YP_002154378.1) and Tp250 (YP_009219585.1), all carrying icosahedron head structures [19, 20]. The MCP of PfEFR-4 (ORF9) showed 82% similarity with that of *Paenibacillus* phage HB10c2 (YP_009195195.1), both carrying a similar elongated head [26], which is a relatively rare morphology for bacterial phages. The MCP (ORF5) of 11143 had 64% similarity with that of vB_BhaS-171 (YP_00927333.1), but no significant similarity with that of the other four compared phages.

The host lysis regions of the five phages were analyzed. The endolysin of PfEFR-4 shared 95% similarity to that of Tp250 and vB_BceS-IEBH, with an amidase 02_C cell wall binding domain (CBD) and a peptidoglycan recognition protein (PGRP) N-terminal catalytic domain (EBD). The endolysin of PfIS075 contained a SH3_3 CBD and PGRP EBD, and the endolysin of 11143 (ORF20) had a putative GH25_PlyB-like EBD and amidase 02_C homologous CBD.

In addition to the common DNA replication proteins of the phages, there was a plasmid-like region, including a potential replication protein and plasmid segregation protein (ParM) in PfIS075 (ORF44-45), with the latter as an actin homologue driving plasmid partition and DNA segregation by polymerizing to filaments [27, 28]. The highly similar plasmid-like region was also presented in Tp250 and vB_BceS-IEBH, which has self-replicating capability *in vivo* [19, 20, 29]. Their existence within the hosts was evidence for these phages as phagemids.

### Phylogeny of 36 *Bacillus* phages

**Fig 5:**
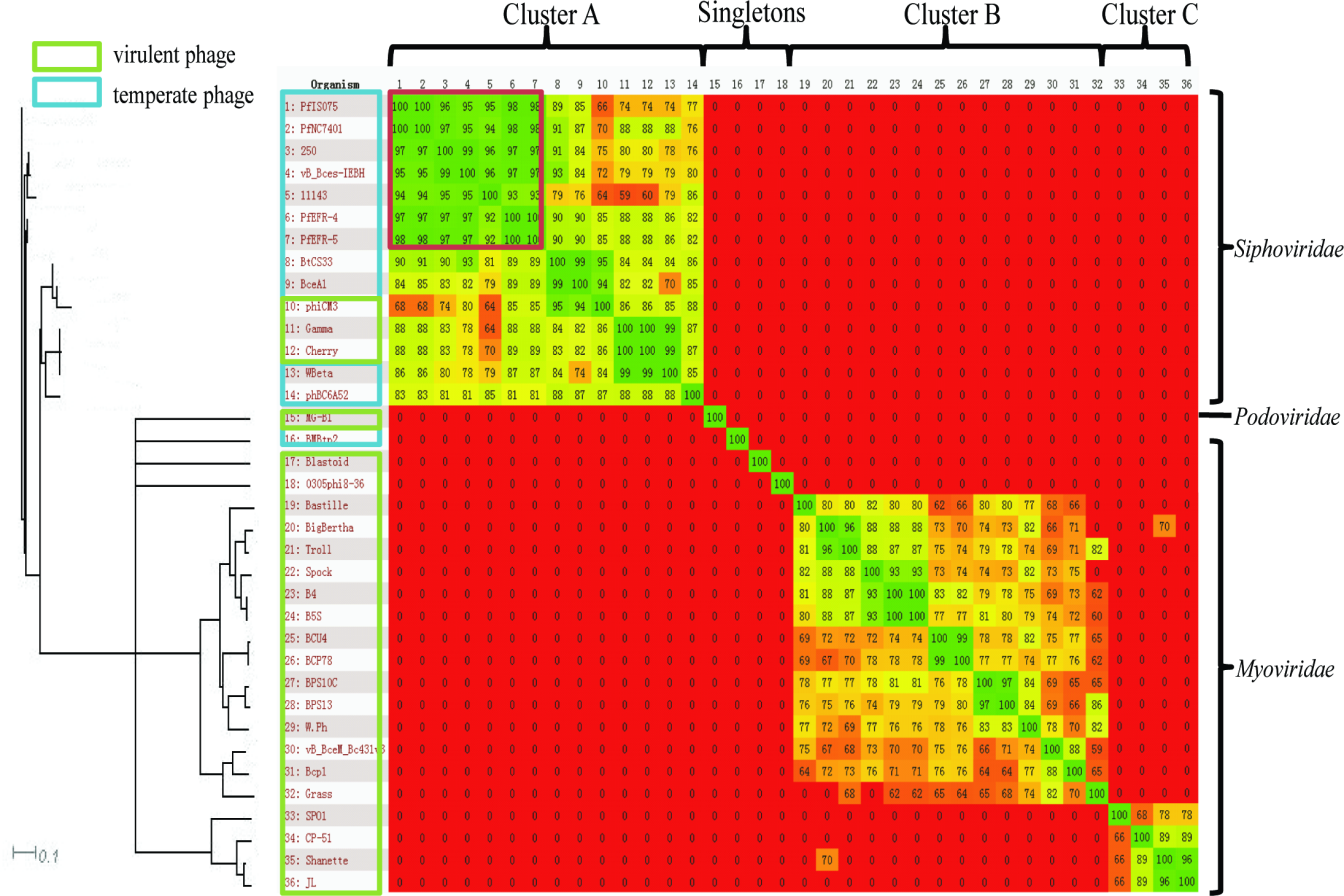
Heat-plot analysis of 36 *Bacillus* phages revealed three clusters and four singletons using Gegenees. Fragmented alignment based on BLASTN was performed with settings 50/25. The cutoff threshold for non-conserved material was 30%. A dendrogram was produced in SplitsTree 4 (using neighbor joining) made from a Nexus file exported from Gegenees.

The genomes of 36 phages derived from *Bacillus* spp., listed in S3 Table with their GenBank accession numbers and basic phage properties, were aligned and compared using Gegenees [30]. The heat-plot based on the nucleotide alignment revealed three clusters (A-C) and four singletons (Fig 5). Except for phBC6A52 (*Podoviridae*), cluster A consisted of 14 phages, belonging to *Siphoviridae* with genome sizes varying from 37.4 kbp to 56.5 kbp. Within this cluster, the seven phages derived from emetic *B*. *cereus* (PfIS075, PfNC7401, vB_BceS-IEBH, Tp250, 11143, PfEFR-4, and PfEFR-5) had more than 92% similarity with each other; Gamma and Cherry, phages successfully used for the rapid clinical diagnosis of *B*. *anthracis*, showed 99% similarity with the temperate phage Wbeta derived from *B*. *cereus*. Cluster B consisted of 14 phages, including nine *B*. *cereus*, three *Bacillus thuringiensis*, one *B*. *anthracis*, and one *Bacillus subtilis* derived phages, all of which were virulent myoviruses carrying the largest genomic DNA from 152.8 kbp to 163.0 kbp. Cluster C was comprised of four *Myoviridae* phages derived from *B*. *subtilis* and *B*. *cereus* with medium genomes (132.6−138.9 kbp). For the singletons, MG-B1 and Blastoid were *B*. *weihenstephanensis* and *Bacillus pumilus* virulent phages, belonging to *Podoviridae* and *Siphoviridae* with genomes of 27.19 kbp and 50.354 kbp, respectively; BMBtp2 and 0305Φ8–36 were derived from *B*. *thuringiensis*, with the former a temperate phage with a genomic DNA of 36.932 kbp, belonging to *Siphoviridae*, and the latter an atypical jumbo myovirus carrying a giant genome of 218.948 kbp.

**Fig 6:**
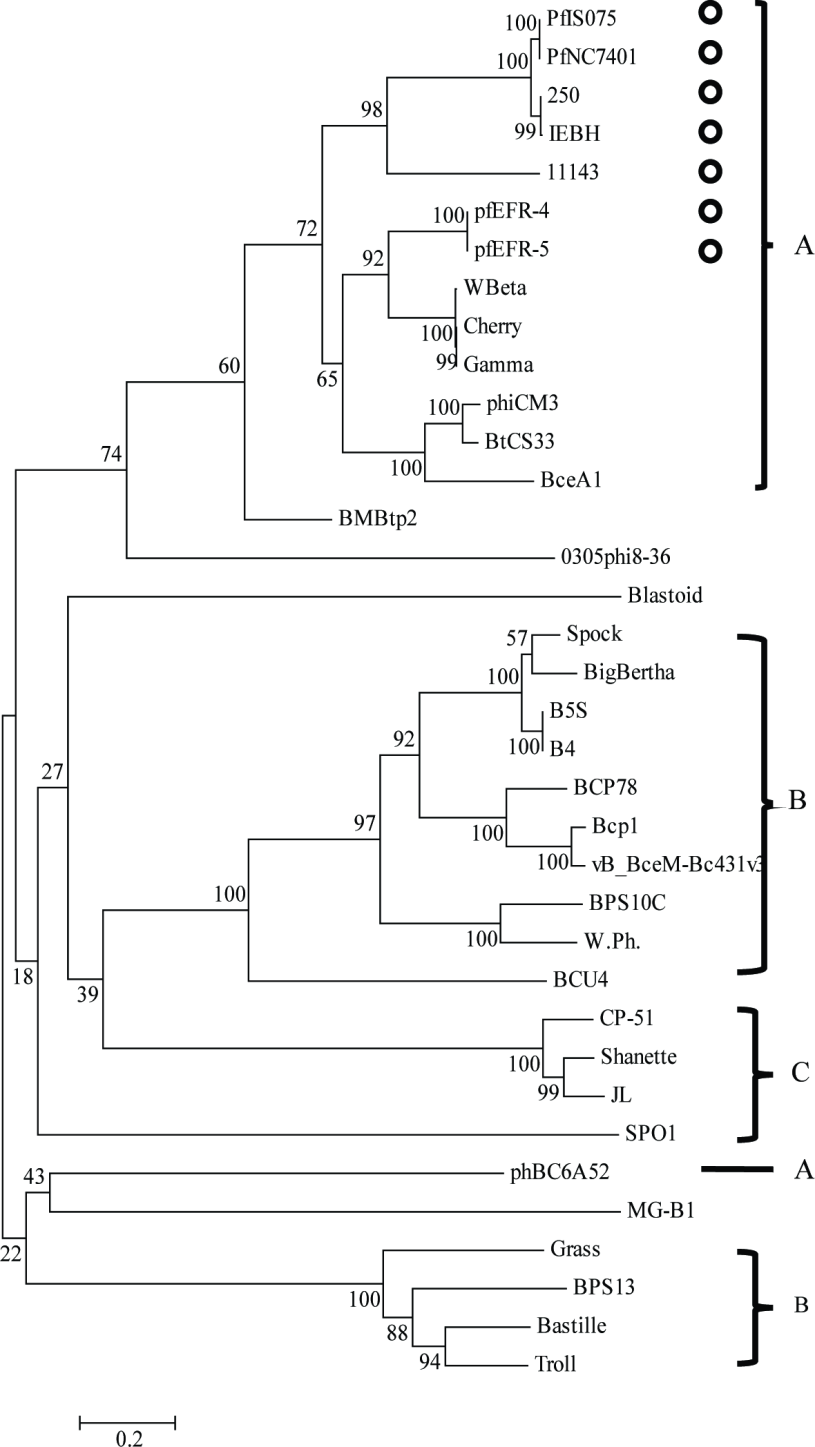
Phylogenetic tree based on the tail fiber proteins of 36 *Bacillus* phages. Phylogenetic trees were constructed based on the amino acid sequences of the tail fiber proteins of 36 *Bacillus* phages using neighbor joining with a bootstrap of 1,000. Circles represent the phages derived from emetic isolates. Numbers on the lines indicate branch support. Corresponding clusters (A-C) are also shown on the right.

Furthermore, comparative phylogenetic analysis using tail fiber proteins (Fig 6), which are a crucial part of phages initiating absorption and infection to the host [31–36], displayed similar classification to that based on the genome sequences of the derived phages. Remarkably, phages derived from the emetic isolates shared conserved tail fiber proteins and similar genome structures, corresponding to their host spectrum specificity.

### Cloning and characterization of lysins *in vitro*

**Fig 7:**
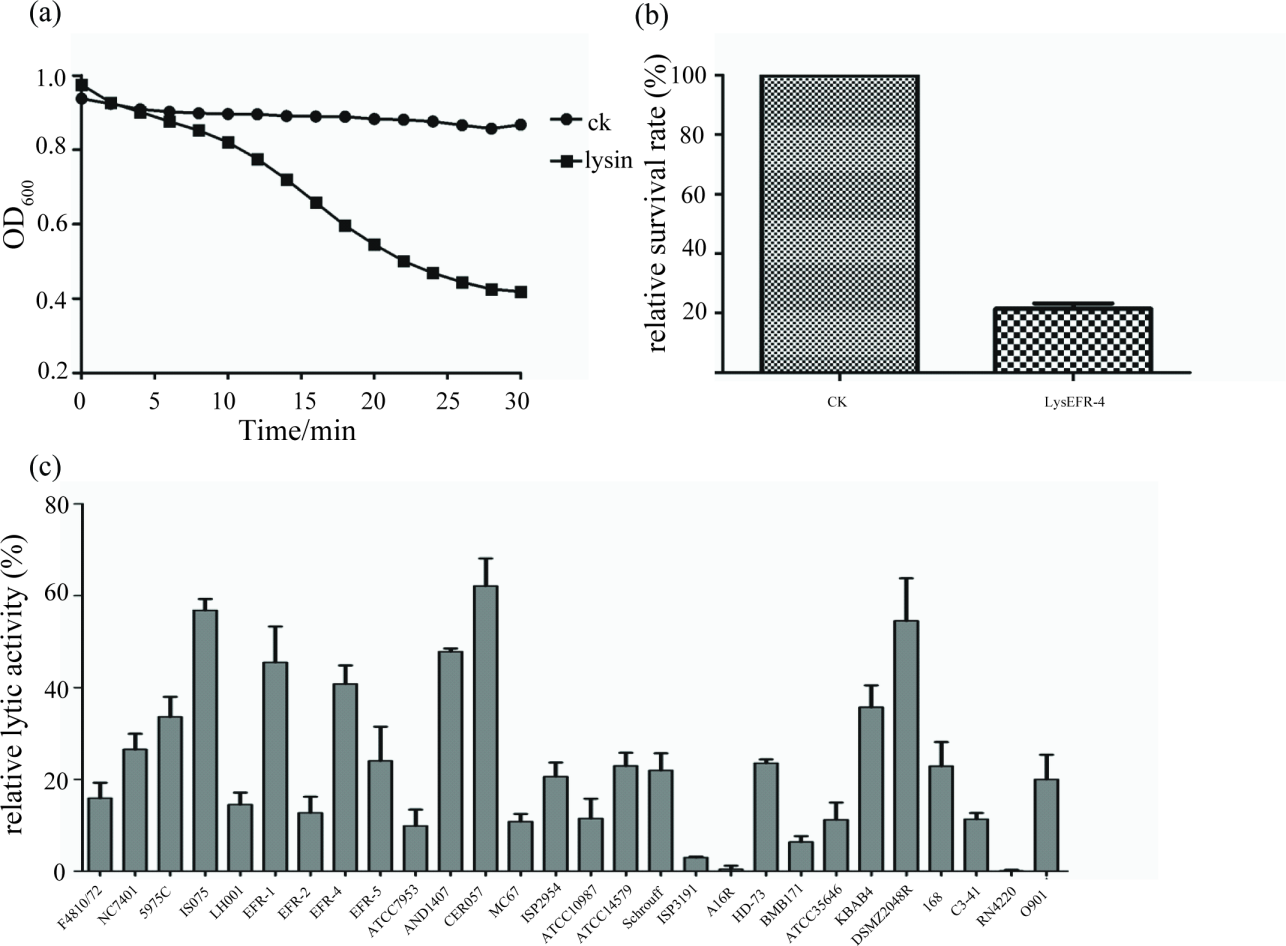
Lytic activity and antimicrobial spectrum of LysEFR-4. Lytic activity of LysEFR-4 was measured by (a) turbid reduction assay and (b) plate lysis assay. CK indicates the control with the same volume of dialysis buffer replacing LysEFR-4 in the assay. (c) Antimicrobial spectrum of LysEFR-4. All experiments were carried out in triplicate.

The predicted lysins PfEFR-4-ORF24 (named LysEFR-4) and PfNC7401-ORF49 (named LysNC7401) were cloned and expressed heterologously. The purified recombinant protein LysEFR-4 was visualized as a clear protein band of ca. 32 kDa on SDS-PAGE gel, in line with the predicated size (data not shown). Bioinformatics analysis showed that LysEFR-4 carried a PGRP superfamily EBD domain and a putative amidase 02_C CBD domain. LysEFR-4 could significantly lyse the derived emetic bacterial cells, causing a decrease in absorbance at 600 nm due to 60% and 80% cell death (Fig 7A and Fig 7B). In addition, LysEFR-4 could lyse all 15 tested emetic *B*. *cereus* and *B*. *weihenstephanensis* isolates, and could also cross-lyse some other *Bacillus* species strains (Fig 7C).

Although putative *lysNC7401* was cloned into four positive-selection vectors (i.e., pET28a, pQE30, pGEX-6p-1, and pMAL-C2x), after transformation into *E*. *coli* only a few colonies were able to grow. Sequencing these recombinants showed that they all harbored one or several random mutations, which occurred in the active center of the predicted functional domain PGRP, including substrate binding sites (163–168, 223–225, 235–237, 274–276, 295–300, 316–318, 454–456, 466–468, and 490–498), Zn binding residues (160–162, 454–456, and 496–498), amidase catalytic sites (160–162, 235–237, 454–456, 490–492, and 496–498) or frameshift mutations from one or more base deletions. Therefore, LysNC7401 could not be expressed heterologously with success. The possible reason was that the lysin might be toxic to the expression strain [37].

## Discussion

The *B*. *cereus* group members, including *B*. *cereus*, *B*. *thuringiensis*, *B*. *anthracis*, *B*. *weihenstephanensis*, *Bacillus mycoides*, *Bacillus pseudomycoides*, and *Bacillus cytotoxicus*, share similar chromosomal backbones and biochemical characters [38–45]. Their classification is mainly based on distinctive pathotypes and ecotypes, which are determined by the accessory genes exclusively owned by each species. The MGEs (e.g., large plasmids, transposons, insertion sequences, and phages) play important roles in the acquisition of highly specialized accessory genes, and thus some are used for differentiating or typing species. A typical example is *B*. *anthracis*, whose fatal anthrax is genetically determined by its pXO1 and pXO2 plasmids harboring pathogenicity islands with the specific anthrax virulence genes flanked by insertion sequences [46]. Furthermore, as well as the pXO1 and pXO2 plasmids, the *B*. *anthracis* genome contains four putative lambdoid prophages, which can be used for differentiating *B*. *anthracis* from other *B*. *cereus* members [18]. Previous study has shown flexible plasmid content of the emetic *B*. *cereus* group strains [5], and relatedness between the “ces” cluster and transposons [6]. However, phages have received less attention in terms of their potential contribution to distinctive ecotypes and pathotypes. In this study, we focused on the characterization of the phages preying on emetic *B*. *cereus* isolates. This study will enrich our knowledge on bacterial genetic variability and the evolution of emetic *B*. *cereus* group isolates. The phages induced from the five emetic strains all belonged to the *Siphoviridae* family, but presented in different forms in their hosts. PfNC7401 and PfIS075 had typical icosahedral heads and their genetic materials were circular dsDNA containing both phage and plasmid replication genes. Due to their high similarity with vB_BceS-IEBH, whose genome had a plasmid-like region and phagemid state, PfNC7401 and PfIS075 should exist alone in plasmid form in the host, with self-replicating capability in the lysogenic state, and produce active phages through induction. Indeed, the alignment of PfNC7401 to the host genome makes a complete match to one replicon (plasmid) of *B*. *cereus* NC7401. Two other intact prophages (ca. 49−51 kbp) were predicted within the chromosome of *B*. *cereus* NC7401 using PHAST (data not shown), which showed no obvious similarity with PfNC7401 and no induction to active phages in this study. Although no complete genome was available for IS075, the current alignment results indicated that PfIS075 might match to one plasmid replicon of IS075, but not to the other predicted prophages (ca. 48−53 kbp) located in the chromosome (data not shown). PfEFR-4, PfEFR-5, and PfATCC7953 had elongated heads, with the genomes of PfEFR-4 and PfEFR-5 being linear dsDNA, whereas that of PfATCC7953 was undetermined. The replication region of PfEFR-4 and PfEFR-5, containing only DnaB and DnaC and lacking other replication factors, suggests it is not autoreplicated, but integrated into the host genome during the lysogenic state.

Although displaying diverse sizes and DNA forms, the phages derived from emetic *B*. *cereus* isolates showed similar genome structures and core genes. Phylogenic analysis based on the complete genomes and conserved tail fiber proteins of 36 *Bacillus* phages confirmed that the phages derived from emetic *B*. *cereus* strains were highly similar, which corresponded to their host spectrum specificity. This suggests there is a coevolutionary relationship among the prophages and the hosts. Previous study has reported that prophages of tectivirus GIL01 and GIL16 greatly contribute to the adaptation of the host to the environment, and the cholera toxin, which is produced by filamentous phage CTXɸ, endows the host pathogen with cholera virulence [47, 48]. Whether the *Siphoviridae* prophages explored in this study are associated with the hosts’ pathotypes and ecotypes remains unclear, and the host-phage coevolution needs to be further studied.

At the end of their life cycle, the phage endolysin degrades the host cell wall with the help of the membrane protein holin, releasing mature virions [49]. Thus, the lysis spectrum of a phage is related with that of the endolysin, though not exactly. The process under which phages infect bacteria and produce progeny viruses is influenced by the invaders as well as the hosts, which own a series of defense systems such as CRISPER-Cas, restriction-modification, and resistant mutations [50]. However, lysins can directly lyse Gram-positive bacteria *in vitro* due to exposing the peptidoglycan, which is the outermost cell wall structure. Therefore, it is reasonable that the antimicrobial spectrum of endolysin is usually wider than the host range of the phage. The host of PfEFR-4 was restricted to emetic *B*. *cereus*, whereas LysEFR-4 could not only lyse all the tested emetic *B*. *cereus* group isolates, but also showed cross-bactericidal activity against other *Bacillus* strains and Gram-negative pathogen *Salmonella enteric* var. Typhimurium, implying a potential of endolysin as an additional antimicrobial agent combined with other additives to control food contamination. An exhaustive attempt was carried out to clone ORF49 encoding endolysin in PfNC7401 in *E*. *coli*. However, almost all clones screened contained mutations (ca. 95.7% mutation rate) in the predicted active center of the enzyme. Furthermore, after addition of IPTG to the only plasmid without mutation, the *E*. *coli* BL21 became clear. This suggests that the endolysin of PfNC7401 might be toxic or have some lysis activity against *E*. *coli*, and therefore, only the clones with mutations could survive. Even if one correct recombinant plasmid existed by chance, the recombinant bacteria containing the plasmid could not express abundant proteins in the cell [37].

## Materials and methods

### Bacterial strains and growth conditions

The bacterial strains used for phage induction and host spectrum analysis in this study consisted of 24 *B*. *cereus* group isolates isolated from food, animal, clinical, and soil samples, and four from other species. Their characteristics, origins, genome accession numbers, and sources/references are listed in Table 1. The *Bacillus* strains in this study were grown in standard Luria-Bertani (LB) medium at 30°C, and the other strains, including *Staphylococcus aureus* and *S*. Typhimurium, were grown at 37°C.

### Induction of bacteriophages and host range assay

200 μl of overnight bacterial culture was transferred into 50 mL of fresh LB medium to subculture to the logarithmic phase (OD_600_ = 0.2−0.3), after which mitomycin C (Sigma, USA) with a final concentration of 1 μg/mL was added. Bacterial lysis was observed after 1 h of incubation at 30°C. Until the OD_600_ of the bacterial culture did not decrease, the cell lysates were centrifuged for 15 min at 10,000 × g and at 4°C. The supernatant was then passed through a 0.22-μm pore size syringe filter (Millipore, USA) and stored as crude extract of the phage at -80°C and 4°C until use. The host range of the phages were tested with the drop method on 26 *B*. *cereus* group strains and four strains from other species, including *B*. *subtilis* 168, *Lysinibacillus sphaericus* C3-41, *S*. *aureus* RN4220, and *S*. Typhimurium O901. The lysis zones were observed after 10 μL of phage lysates were plotted on soft agar (0.5%) with bacterial lawns and incubated at 30°C overnight.

### Propagation and purification of the phages

Propagation and purification of the phages were performed as per previous methodology [51], with some modifications. The *B*. *cereus* strain most sensitive to infection, as per the spot test under visual observation, was used as the propagation host of the corresponding phage. Accordingly, EFR-4 was used to propagate PfEFR-4, PfEFR-5, and PfATCC7953, EFR-5 was used to propagate PfIS075, and LH001 was used to propagate PfNC7401. The propagation strain with the phage was incubated on double layer plates until plaques formed. One plaque was picked and suspended in 100 μL of SM buffer (50 mM Tris-Cl, pH 7.5; 100 mM NaCl; 10 mM MgSO_4_∙7H_2_O), then mixed with 200 μL of propagation strain culture (OD_600_ ≈ 1.0) and incubated for 30 min at 30°C. Subsequently, the mixtures were added to 5 mL of molten semisolid medium with soft agar (0.5%) kept at 47°C and poured onto solid medium (1.5%) after thorough mixing, then incubated at 30°C overnight. For purification, the phages were passaged at least five times until homogeneous plaques were formed. Then, the purified temperate bacteriophages were propagated using the method described above, except that bacteriophage suspensions with a titer above 10^5^ PFU/mL were used. After plaques were formed, 5 mL of SM buffer was added onto the plate and left at 4°C for 4 h with moderate rotation. The suspension was then recovered and centrifuged at 4°C for 15 min at 10,000 × g. The supernatant was filtered through a 0.22-μm sterile filter, and the phage preparations were stored at -80°C and 4°C until further use.

### Morphological observation of the phages under TEM

The phage preparation was centrifuged at 118,000 × g for 2 h at 4°C (Beckman), and the pellet was resuspended in SM buffer. Then, 20 μL of the phage suspension (10^10−11^ PFU/mL) was immediately deposited onto copper grids with carbon-coated Formvar films for 3−5 min. The grids were then stained with 2% phosphotungstic acid solution (pH = 7) for 3 min and examined with a H-7000FA TEM (Hitachi, Tokyo, Japan) at an acceleration voltage of 75 kV.

### Bacteriophage DNA extraction

Genomic DNA from the phages was extracted according to previous methods [7], with minor modifications. The phage suspension was treated with DNase I (7 U/mL) and RNase A (20 μg/mL) at 37°C for 1 h to remove the bacterial DNA and RNA, respectively. Then, 20 μL of ZnCl_2_ solution (2 M) was added and the solution was incubated at 37°C for 5 min. After centrifugation at room temperature for 1 min at 10,000 × g, the supernatant was removed and the pellet was suspended in 500 μL of TES buffer (0.1 M Tris-Cl, pH 8.0; 0.1 M EDTA; 0.3% SDS) and incubated at 60°C for 15 min for lysis of the phage particles. After the addition of 20 μL of proteinase K (20 mg/mL) and incubation at 37°C for 90 min, 60 μL of 3 M potassium acetate solution (pH 5.2) was completely mixed with the suspension and then kept on ice for 15 min. To remove proteins from the phage suspension, the mixture was treated with phenol/chloroform/isoamyl alcohol (25:24:1, v/v) twice, and then with chloroform/isoamyl alcohol (24:1, v/v) once. Subsequently, phage DNA was precipitated with an equal volume of isopropanol and washed with 70% ethanol, and finally dissolved in 10−20 μL of distilled water. The genomic DNA isolated from the phages was examined by 0.6% agarose-gel electrophoresis.

### DNA restriction and structural protein analysis

One microgram of phage DNA was digested for 3 h at 37°C with *Eco*RI, *Bam*HI, and *Pst*I (Takara), respectively, in appropriate restriction buffer. The digested products were then analyzed by 0.6% agarose-gel electrophoresis. The structural proteins of the five purified phages were preliminarily visualized by 15% SDS-PAGE. To better illustrate the proteomics of the phages, PfIS075 and PfEFR-4 were boiled for 10 min and then underwent 10% SDS-PAGE. After the samples were fully condensed and the bromophenol blue had migrated about 1 cm in the separating gel at 80 V for 1.5 h, the gel containing all the proteins was cut, reduced, alkylated, and trypsin-digested for peptide LC-MS/MS identification, as reported previously [20].

### Genome sequencing and bioinformatics analysis

The genome sequencing of the phages was performed by BGI Co. (Wuhan, China) with Illumina HiSeq 2000 (Illumina, San Diego, CA, USA) sequencing. The genomes were assembled using SOAPdenovo software version 2.04 (http://sourceforge.net/projects/soapdenovo2/files/SOAPdenovo2/). The remaining gaps between the scaffolds of the phages were filled using polymerase chain reaction (PCR). The coding sequences (CDSs) were predicted with Glimmer 3.02 (http://www.cbcb.umd.edu/software/glimmer/), FGENE SV (http://linux1.softberry.com/berry.phtml?topic=virus&group=programs&subgroup=gfindv), and PHAST (http://phast.wishartlab.com) [52–55]. ATG, GTG, and TTG were used as possible start codons. The putative function of each gene was predicted with the Gene Ontology (GO), Kyoto Encyclopedia of Genes and Genomes (KEGG), Swiss-Prot, Cluster of Orthologous Groups of proteins (COG), and Non-Redundant Protein databases, with the best matches chosen to be the functional annotation of the genes. The molecular weights and isoelectric points were calculated using Compute pI/Mw (http://www.expasy.ch/tools/pi_tool.html). tRNAscan-SE 1.21 (http://gtrnadb.ucsc.edu/), RNAmmer 1.2 (http://www.cbs.dtu.dk/services/RNAmmer/), and Rfam 12.0 (http://rfam.sanger.ac.uk/) were used to search for the genes encoding putative tRNA, rRNA, and sRNA, respectively [56–58]. Tandem repeat and insert sequences in the phages were analyzed using Tandem Repeat Finder 4.08 (http://tandem.bu.edu/trf/trf.html) and ISfinder (https://www-is.biotoul.fr/search.php), respectively [59, 60]. Promoter sequences and oligonucleotides from known transcription factor binding sites were identified using Bprom (http://linux1.softberry.com/berry.phtml). Comparative genome analysis of the phages at the nucleotide level was conducted using Gegenees 2.1 (window size of 50 bp, step-size of 25 bp, and cutoff of 30%) and Mauve, and comparison at the proteomic level was made using CoreGenes 3.0 (http://binf.gmu.edu:8080/CoreGenes3.0). Genes with scores above 75 were regarded as core genes [22, 30, 61]. The protein sequences of the tail fibers used for alignment were available from the protein database http://www.ncbi.nlm.nih.gov/protein, and alignment was carried out by CLUSTAL W [62]. The phylogenetic tree in this study was constructed using Mega 6 software with a bootstrap of 1,000 [63]. The GenBank accession numbers of the seven phages derived from emetic *B*. *cereus* used in this study were GU229986 for Tp250, NC_011167 for vB_BceS-IEBH, GU233956 for 11143, KX227757 for PfEFR4, KX227758 for PfNC7401, KX227759 for PfIS075, and KX227760 for PfEFR-5.

### Cloning, lytic activity, and antimicrobial spectra of endolysin

The predicted lysin of EFR-4, ORF24, was amplified using primers lysEFR-4F (5'-CCGGAATTCATGGAAATTAAACAAATGTTAGTAC-3') and lysEFR-4R (5'- CCGCTCGAGCTATTCTTTTGTATAGAAGTATTTA-3'). The 756 bp purified PCR product was digested with *Eco*RI/*Xho*I and cloned into pET28a. The putative endolysin of PfNC7401, ORF49 (1,065 bp), was amplified using primers lysNC7401F (5'-ATGAAAAAAACTTTTAAACTGGCTT-3') and lysNC7401R (5'-TTACTTCACATACACATAGGCTTCA-3'), with different enzymes *Eco*RI/*Xho*I, *Kpn*I/*Hind*III, *Eco*RI/*Xho*I, and *Eco*RI/*Pst*I digested and cloned into the expression vectors pET28a, pQE30, pGEX-6p-1, and pMAL-C2x, respectively. The recombinant protein was induced to be expressed in *E*. *coli* BL21 with 1 mM isopropyl-ß-d-thiogalactoside (IPTG) at 30°C for 5 h and purified with an Ni-NTA column (Roche, Basel, Switzerland). The purified endolysin was visualized with 15% SDS-PAGE and dialyzed against the buffer (20 mM Tris-Cl, pH 8.0, 10% glycerol, 1mM EDTA) and stored at -80°C till use.

The lytic activity of LysEFR-4 was tested by turbid reduction and plate lysis assays [64–66]. In brief, *B*. *cereus* EFR-4 was grown to the logarithmic phase at 30°C in LB medium, and then harvested by centrifugation at 10,000 × g for 1 min at room temperature and resuspended in 20 mM Tris-Cl (pH 9.0) to adjust the OD_600_ to 0.8−1.0. We then added 0.6 μM LysEFR-4 to the bacterial suspension, with an equal volume of dialysis buffer added to the control. Lytic activity measured using the turbid reduction assay was then monitored by the decrease in absorbance at OD_600_ at 37°C for 30 min with a multimode reader (Bio-Tek Synergy HT, Winooski, VT, USA), whereas the lytic activity using the plate lysis assay was measured via the relative bacteria survival rate at 37°C for 1 h by counting the growing colonies (CFU/mL) of the mixture with bacterial cells and endolysin divided by that of the control without endolysin. To determine the antimicrobial spectrum of the endolysin, we measured lytic activity monitored by turbid reduction on 28 bacteria, including *B*. *cereus* group isolates (covering *B*. *cereus*, *B*. *thuringiensis*, *B*. *anthracis*, *B*. *weihenstephanensis*, and *B*. *mycoides*), *B*. *subtilis*, *L*. *sphaericus*, *S*. *aureus*, and *S*. Typhimurium. When treating the Gram-negative bacterium, 0.1 M EDTA was added to penetrate the outer membrane to access the peptidoglycan of the cell wall.

## Supporting information

S1 Table Genome overview of the four isolated phages. (DOCX)

S2 Table Primers used for verification of circularity or linearity of the phage genomes. (DOCX)

S3 Table Genomic features of the phages used in this study. (DOCX)

S1 Fig Prophage induction curves of the five emetic *B*. *cereus* strains by mitomycin C. Prophage induction of NC7401, IS075, EFR-4, EFR-5, and ATCC7953 was conducted with 1 μg/mL of MMC. Dashed lines indicate growth curves with induction, solid lines represent the control without induction.
(TIF)

S2 Fig Digestion profiles of the phage genomes. Phage DNA of PfIS075, PfNC7401, PfEFR-5, PfEFR-4, and PfATCC7953 was digested with restriction enzymes *Eco*RI, *Bam*HI, and *Pst*I and checked with 0.6% agarose gel electrophoresis. Lane M: Trans15K DNA marker; Lane 1−3: PfIS075- *Eco*RI/ *Bam*HI/ *Pst*I; Lane 4−6: PfNC7401- *Eco*RI/ *Bam*HI/ *Pst*I; Lane 7−9: PfEFR-5- *Eco*RI/ *Bam*HI/ *Pst*I; Lane 10−12: PfIEFR-4- *Eco*RI/ *Bam*HI/ *Pst*I; Lane 13−15: PfATCC7953- *Eco*RI/ *Bam*HI/ *Pst*I.
(TIF)

S3 Fig Verification of genome status of the four sequenced phages. Lane M: Trans2K PlusII DNA marker; four pairs of primers PFNG-F/R, PFIG-F/R, PF4G-F/R, and PF5G-F/R were used for verification of the circularity or linearity of genomes of phages PfNC7401, PfIS075, PfEFR-4, and PfEFR-5, respectively.
(TIF)
