# Supplementary material for: Identification and genomic comparison of temperate bacteriophages derived from emetic Bacillus cereus
Source: PLoS One. 2017 Sep 8;12(9):e0184572. doi: 10.1371/journal.pone.0184572 (PMC5590980; doi:10.1371/journal.pone.0184572)
Supplement: S1 Table — (DOCX) [file pone.0184572.s001.docx]

|  | PfIS075 | PfNC7401 | PfEFR-4 | PfEFR-5 |
| --- | --- | --- | --- | --- |
| Genome size(bp) | 48,626 | 47,972 | 43,223 | 43,773 |
| GC Content(%) | 36.49 | 36.54 | 35.44 | 35.39 |
| Gene Number | 81 | 79 | 67 | 69 |
| Gene Length/Genome(%) | 84.33 | 84.08 | 85.01 | 84.85 |
| Tandem Repeat Number | 4 | 4 | 8 | 6 |
| Tandem Repeat Length/Genome(%) | 0.42 | 0.40 | 1.22 | 0.70 |
| Minisatellite DNA Number | 3 | 3 | 8 | 6 |
| Microsatellite DNA Number | 1 | 1 | 0 | 0 |
| IS Number | 18 | 18 | 16 | 15 |
| rRNA Number | 0 | 0 | 0 | 0 |
| tRNA Number | 0 | 0 | 0 | 0 |
| sRNA Number | 0 | 0 | 0 | 0 |

Table S2. The genome overview of the four isolated phages.
