# Supplementary material for: Identification and genomic comparison of temperate bacteriophages derived from emetic Bacillus cereus
Source: PLoS One. 2017 Sep 8;12(9):e0184572. doi: 10.1371/journal.pone.0184572 (PMC5590980; doi:10.1371/journal.pone.0184572)
Supplement: S2 Table — (DOCX) [file pone.0184572.s002.docx]

Table S3. The primers used for verification of circularity or linearity of the phages genomes.

| Phage | Primer | Sequence |
| --- | --- | --- |
| PfNC7401 | PFNG-F | 5' GTAGGAATAAACGCTGGAA 3' |
|  | PFNG-R | 5' AGCCCACTTAACTTCTTCAC 3' |
| PfIS075 | PFIG-F | 5' CGTAAGGGTGAAGCATTT 3' |
|  | PFIG-R | 5' AAGTTTCATCCGTTCGTT 3' |
| PfEFR-4 | PF4G-F | 5' CGTTGCCAAGAGTGTATG 3' |
|  | PF4G-R | 5' ACCCGAATGGATGCTTTA 3' |
| PfEFR-5 | PF5G-F | 5' TATCGGCTACTTTCTACTGT 3' |
|  | PF5G-R | 5' CTCTTCGCTTGAATAACAT 3' |
