# Supplementary material for: Identification and genomic comparison of temperate bacteriophages derived from emetic Bacillus cereus
Source: PLoS One. 2017 Sep 8;12(9):e0184572. doi: 10.1371/journal.pone.0184572 (PMC5590980; doi:10.1371/journal.pone.0184572)
Supplement: S3 Table — (DOCX) [file pone.0184572.s003.docx]

Table S1. Genomic features of the phages used in this study.

| **Phage** | **Morphology** | **Host** | **Genome Size (bp)** | **Lifestyle** | **GenBank** **Accession No.** | **Tail fiber** |
| --- | --- | --- | --- | --- | --- | --- |
| PfIS075 | *Siphoviridae* | *B. cereus* | 48,709 | Temperate | KX227759.1 | [ANT40319.1](https://www.ncbi.nlm.nih.gov/protein/1043841591) |
| PfNC7401 | *Siphoviridae* | *B. cereus* | 48,055 | Temperate | KX227758 | [ANT40250.1](https://www.ncbi.nlm.nih.gov/protein/1043841521) |
| PfEFR-4 | *Siphoviridae* | *B. cereus* | 43,223 | Temperate | KX227757 | [ANT40208.1](https://www.ncbi.nlm.nih.gov/protein/1043841478) |
| PfEFR-5 | *Siphoviridae* | *B. cereus* | 43,773 | Temperate | KX227760 | [ANT40387.1](https://www.ncbi.nlm.nih.gov/protein/1043841660) |
| 0305ϕ8-36 | *Myoviridae* | *B. thuringiensis* | 218,948 | Virulent | EF583821 | YP_001429639.1 |
| B4 | *Myoviridae* | *B. cereus* | 162,596 | Virulent | JN790865 | AEZ66040.1 |
| B5S | *Myoviridae* | *B. cereus* | 162,598 | Virulent | JN797796 | AEW47476.1 |
| Bastille | *Myoviridae* | *B. cereus* | 153,962 | Virulent | JF966203 | YP_006907332.1 |
| BCP78 | *Myoviridae* | *B. cereus* | 156,176 | Virulent | JN797797 | AEW47211.1 |
| BCU4 | *Myoviridae* | *B. cereus* | 154,371 | Virulent | JN797798 | AEW47696.1 |
| BigBertha | *Myoviridae* | *B. thuringiensis* | 162,661 | Virulent | KF669647 | AGY46601.1 |
| BPS10C | *Myoviridae* | *B. cereus* | 159,590 | Virulent | KC430106 | AGI12243.1 |
| BPS13 | *Myoviridae* | *B. cereus* | 158,305 | Virulent | JN654439 | AEZ50415.1 |
| JL | *Myoviridae* | *B. cereus* | 137,918 | Virulent | KC595512 | AGR46787.1 |
| Shanette | *Myoviridae* | *B. cereus* | 138,877 | Virulent | KC595513 | AGR47009.1 |
| Spock | *Myoviridae* | *B. thuringiensis* | 161,497 | Virulent | KF669662 | AGY48493.1 |
| Troll | *Myoviridae* | *B. thuringiensis* | 163,019 | Virulent | KF208639 | AGT13391.1 |
| vB_BceM_Bc431v3 | *Myoviridae* | *B. cereus* | 158,621 | Virulent | JX094431 | AFQ96524.1 |
| W.Ph. | *Myoviridae* | *B. cereus* | 156,897 | Virulent | HM144387 | YP_004957046.1 |
| 11143 | *Siphoviridae* | *B. cereus* | 39,077 | Temperate | GU233956 | ADA84945.1 |
| Tp250 | *Siphoviridae* | *B. cereus* | 56,505 | Temperate | GU229986 | ADB28369.1 |
| BceA1 | *Siphoviridae* | *B. cereus* | 42,932 | Temperate | HE614282 | CCE73853.1 |
| BMBtp2 | *Siphoviridae* | *B. thuringiensis* | 36,932 | Temperate | JX887877 | YP_007236359.1 |
| BtCS33 | *Siphoviridae* | *B. thuringiensis* | 41,992 | Temperate | JN191664 | AFL46404.1 |
| Cherry | *Siphoviridae* | *B. anthracis* | 36,615 | Virulent | DQ222851 | ABA46394.1 |
| Gamma^a^ | *Siphoviridae* | *B. anthracis* | 37,373 | Virulent | DQ289556 | ABA46504.1 |
| phiCM3 | *Siphoviridae* | *B. thuringiensis* | 38,772 | Virulent | KF296718 | AGV99446.1 |
| vB_BceS-IEBH | *Siphoviridae* | *B. cereus* | 53,104 | Temperate | EU874396 | YP_002154388.1 |
| WBeta | *Siphoviridae* | *B. cereus* | 40,867 | Temperate | DQ289555 | YP_459978.1 |
| MG-B1 | *Podoviridae* | *B. weihenstephanensis* | 27,190 | Virulent | KC685370 | AGI10618.1 |
| phBC6A52 | *Podoviridae* | *B. cereus* | 38,472 | Temperate | NC_004821 | NP_852603.1 |
| CP-51 | *Myoviridae* | *B. cereus* | 138,658 | Virulent | NC_025423.1 | YP_009099084.1 |
| Bcp1 | *Myoviridae* | *B. anthracis* | 152,778 | Virulent | NC_024137.1 | YP_009031370.1 |
| Blastoid | *Siphoviridae* | *B. pumilus* | 50,354 | Virulent | NC_022773.1 | YP_008771851.1 |
| Grass | *Myoviridae* | *B. subtilis* | 156,648 | Virulent | NC_022771.1 | YP_008771463.1 |
| SPO1 | *Myoviridae* | *B. subtilis* | 132,562 | Virulent | NC_011421.1 | YP_002300337.1 |

^a^ Gamma isolate d’Herelle.
