# Supplementary figures and images for: Identification and genomic comparison of temperate bacteriophages derived from emetic Bacillus cereus
Source: PLoS One. 2017 Sep 8;12(9):e0184572. doi: 10.1371/journal.pone.0184572 (PMC5590980; doi:10.1371/journal.pone.0184572)

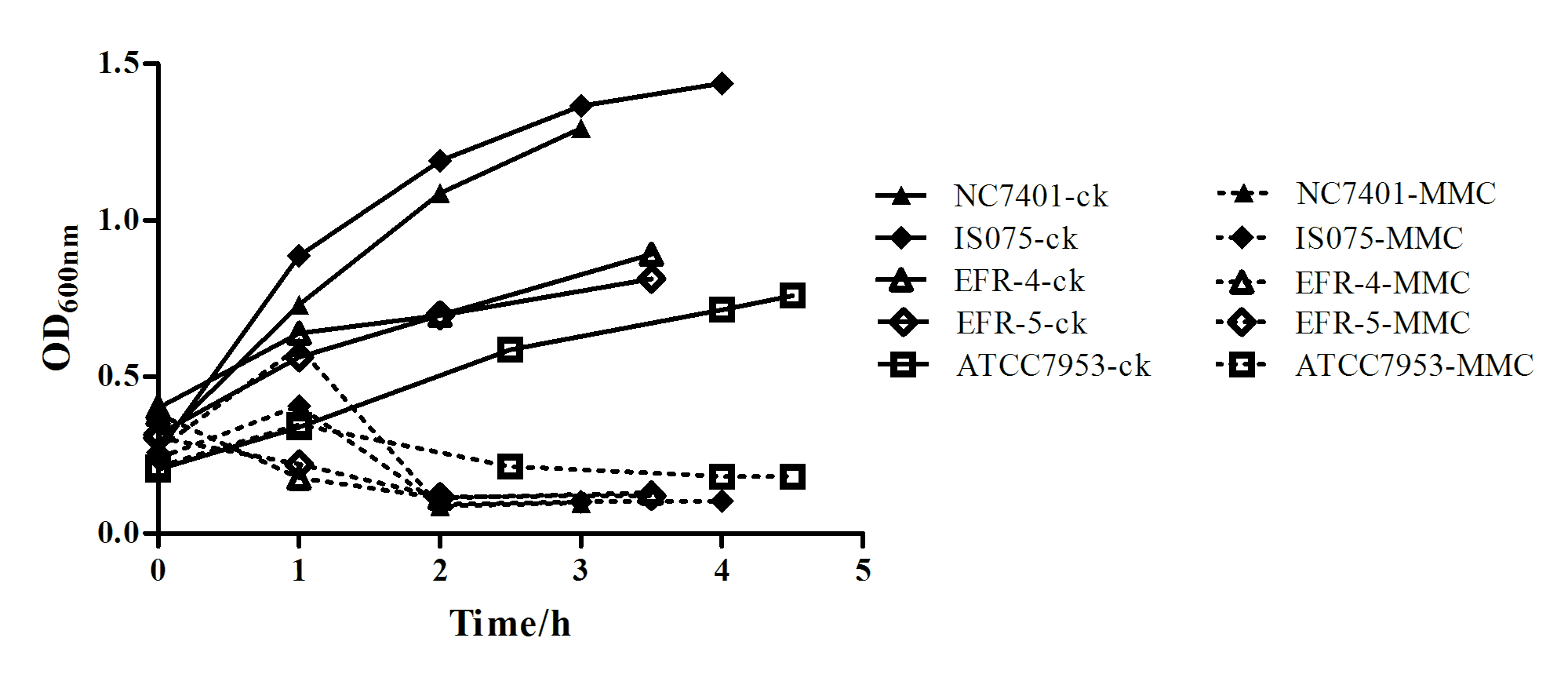

Supplement: S1 Fig — Prophage induction of NC7401, IS075, EFR-4, EFR-5, and ATCC7953 was conducted with 1 μg/mL of MMC. Dashed lines indicate growth curves with induction, solid lines represent the control without induction. (TIF) [file pone.0184572.s004.tif]

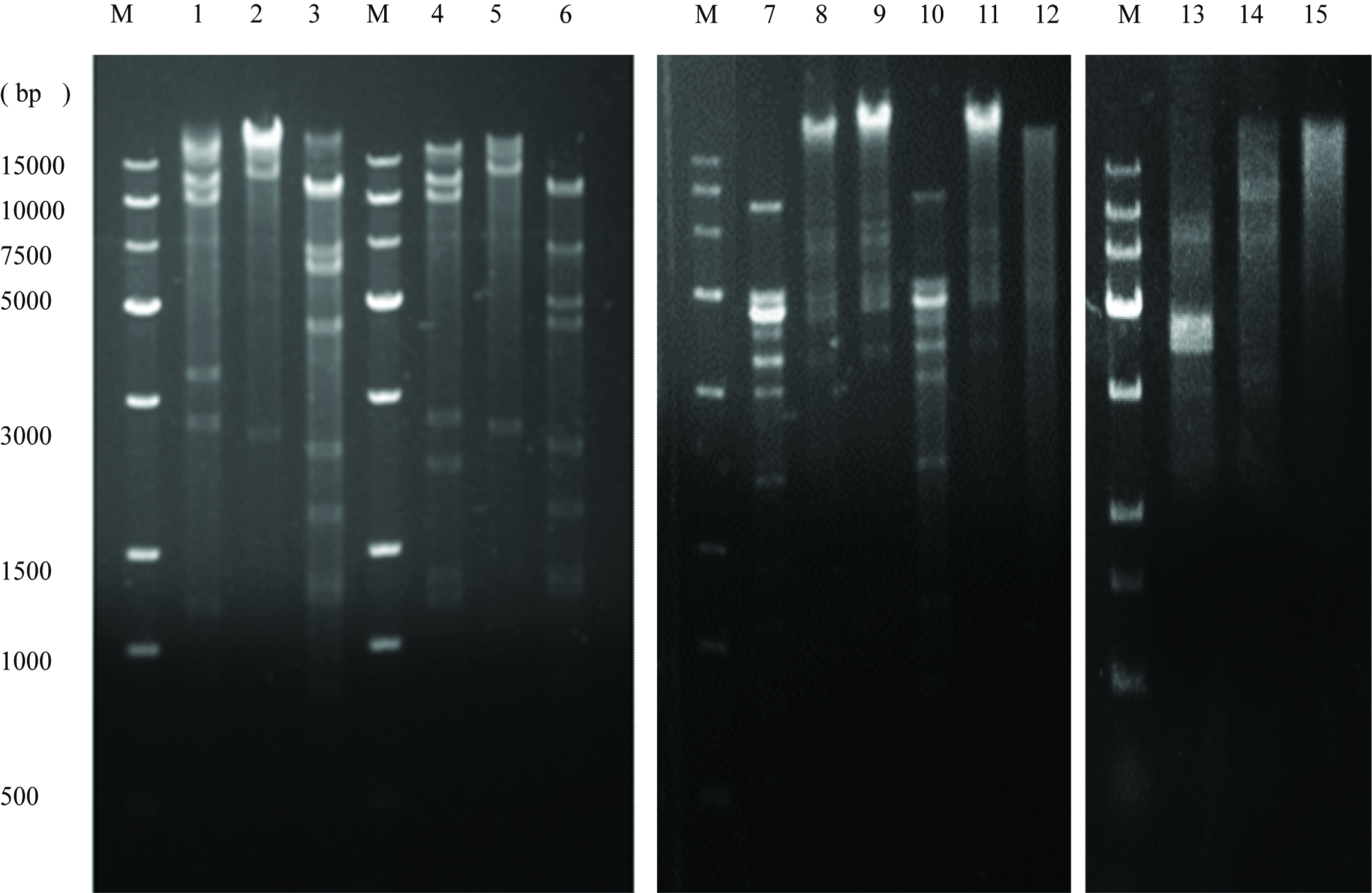

Supplement: S2 Fig — Phage DNA of PfIS075, PfNC7401, PfEFR-5, PfEFR-4, and PfATCC7953 was digested with restriction enzymes EcoRI, BamHI, and PstI and checked with 0.6% agarose gel electrophoresis. Lane M: Trans15K DNA marker; Lane 1−3: PfIS075- EcoRI/ BamHI/ PstI; Lane 4−6: PfNC7401- EcoRI/ BamHI/ PstI; Lane 7−9: PfEFR-5- EcoRI/ BamHI/ PstI; Lane 10−12: PfIEFR-4- EcoRI/ BamHI/ PstI; Lane 13−15: PfATCC7953- EcoRI/ BamHI/ PstI. (TIF) [file pone.0184572.s005.tif]

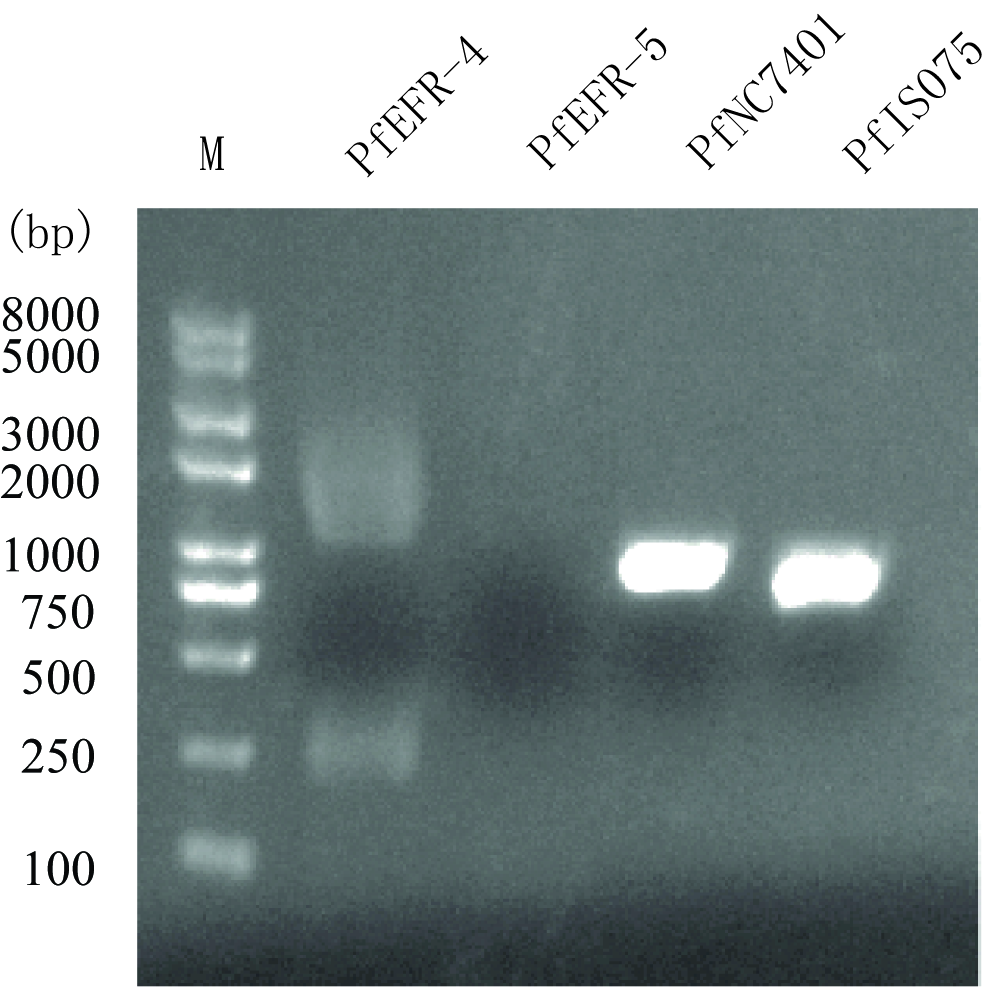

Supplement: S3 Fig — Lane M: Trans2K PlusII DNA marker; four pairs of primers PFNG-F/R, PFIG-F/R, PF4G-F/R, and PF5G-F/R were used for verification of the circularity or linearity of genomes of phages PfNC7401, PfIS075, PfEFR-4, and PfEFR-5, respectively. (TIF) [file pone.0184572.s006.tif]
